# A comprehensive prognostic and immune analysis of *LAPTM4B* in pan-cancer and Philadelphia chromosome-positive acute lymphoblastic leukemia

**DOI:** 10.3389/fimmu.2025.1522293

**Published:** 2025-02-28

**Authors:** Hui Zhou, Yuyao Yi, Wei He, Li Zheng, Yiguo Hu, Ting Niu

**Affiliations:** ^1^ Department of Hematology, West China Hospital, Sichuan University, Chengdu, Sichuan, China; ^2^ Clinic Trial Center, West China Hospital, Sichuan University, Chengdu, Sichuan, China; ^3^ Department of Thyroid Surgery, National Clinical Research Center for Geriatrics, West China Hospital, Sichuan University, Chengdu, Sichuan, China; ^4^ State Key Laboratory of Biotherapy and Cancer Center, West China Hospital, Sichuan University, and Collaborative Innovation Center for Biotherapy, Chengdu, Sichuan, China

**Keywords:** LAPTM4B, Ph+ B-ALL, pan-cancer, diagnosis, prognosis

## Abstract

**Introduction:**

Lysosomal-associated protein transmembrane-4 beta (LAPTM4B) protein expression was increased in solid tumors, whereas few studies were performed in hematologic malignancies. We aimed to study the effect of the LAPTM4B gene in pan-cancer and Philadelphia chromosome-positive acute B cell lymphoblastic leukemia (Ph+ B-ALL).

**Methods:**

The differential expression, diagnosis, prognosis, genetic and epigenetic alterations, tumor microenvironment, stemness, immune infiltration cells, function enrichment, single-cell analysis, and drug response across cancers were conducted based on multiple computational tools. Additionally, Ph+ B-ALL transgenic mouse model with Laptm4b knockout was used to analyze the function of LAPTM4B *in vivo*. BrdU incorporation method, flow cytometry, and Witte-lock Witte culture were used to evaluate the roles of LAPTM4B *in vitro*.

**Results:**

We identified that LAPTM4B expression was increased in various cancers, with significant associations with clinical outcomes. LAPTM4B expression correlated with DNA and RNA methylation patterns and was associated with drug resistance. It also influenced the tumor immune microenvironment, with implications for immunotherapy response. In leukemia, LAPTM4B was expressed in stem cells and associated with specific subtypes. Knockout of LAPTM4B impeded B-ALL progression in mice and reduced cell proliferation and caused G0/G1 arrest *in vitro*.

**Discussion:**

Our study elucidated the role LAPTM4B that promoted the development and progression in Ph+ B-ALL. Furthermore, LAPTM4B played a diagnostic, prognostic, and immunological factor.

## Introduction

Tumorigenesis is a multifaceted process influenced by a dynamic interplay between internal factors and the tumor microenvironment. Internal factors encompass genetic mutations, epigenetic changes, and the dysregulation of signaling pathways (1, 2). Tumor microenvironment is comprised of various factors including metabolomics, inflammation, angiogenesis, immune system modulation, extracellular matrix (ECM) (3–7). Crucially, membrane proteins play a pivotal role during tumorigenesis by facilitating the transmission of signals between the extracellular environment and the cell’s interior (8). These proteins participate some fundamental cellular processes such as growth, differentiation, and survival (8–10). Therefore, investigating the intricate interplay between internal factors, the tumor microenvironment, and the role of membrane proteins is imperative for comprehending the intricacies of tumorigenesis. Such understanding lays the foundation for the development of targeted strategies aimed at preventing and treating cancer effectively. One of the research interests in our laboratory is to uncover the roles and mechanisms of membrane proteins in the initiation and development of tumors. Building upon the reported biological functions of lysosomal membrane-associated protein transmembrane-4 beta (*LAPTM4B*) in existing studies, we aim to comprehensively understand its involvement in cancer, especially Philadelphia chromosome-positive acute B cell lymphoblastic leukemia (Ph^+^ B-ALL).

*LAPTM4B* is recognized as a late endosomal protein, and it is also distributed in the plasma membrane. It exhibits widespread expression in various tissues throughout the body, with predominant levels observed in the heart, kidney, skeletal muscle, and hematopoietic stem cells (HSCs). In contrast, its expression is relatively lower in peripheral blood leukocytes, spleen, and thymus (11). *LAPTM4B* involves in multiple biological processes, including cell cycle, cell growth and proliferation, and autophagy. *LAPTM4B* interacts with and integrin and promotes cell growth and proliferation through a series of enzyme-linked reactions within the membrane (12). *LAPTM4B* also regulates cell cycle and engages in growth signaling pathways, such as PI3K/AKT and MAPK (13). *LAPTM4B* also promotes autophagy through the EGFR signaling pathway (13, 14), and loss of *LAPTM4B* inhibited later stages of autophagy by blocking maturation of the autophagosome (15).

Increased LAPTM4B expression has been observed in various cancers, including breast, liver, lung, ovarian, uterine, and gastric cancers (11, 16–18). Notably, elevated LAPTM4B levels contribute to chemotherapy resistance in breast cancer. The overexpression of LAPTM4B induces resistance to anthracyclines (such as doxorubicin, daunorubicin, and epirubicin) by retaining the drug in the cytoplasm and reducing its nuclear localization, thereby diminishing drug-induced DNA damage (19). In addition to solid tumors, LAPTM4B is also highly expressed in hematologic malignancies. LAPTM4B promoted AML progression by regulating the RPS9/STAT3 axis (20). Elevated LAPTM4B expression is associated with AML patients harboring NPM1 mutations in conjunction with FLT3-ITD mutations (21). In chronic myeloid leukemia (CML) bone marrow (BM) cells, LAPTM4B expression levels were significantly higher than those in normal individuals (22). Similar to observations in solid tumors, CML patients with higher LAPTM4B expression were associated with resistance to tyrosine kinase inhibitor (TKI) treatment (23).

Most studies on LAPTM4B have primarily focused on intracellular signaling in certain types of cancers, and a comprehensive understanding of LAPTM4B in tumorigenesis is still lacking. In this study, we aimed to elucidate the expression, clinical characteristics and immunological characteristics of *LAPTM4B* across various cancers. In particular, our investigation unveiled a significant correlation between *LAPTM4B* expression and survival outcomes in Ph^+^ B-ALL patients. Moreover, it was confirmed that the loss of the *Laptm4b* impeded BCR-ABL-induced B-ALL progression, both *in vitro* and *in vivo*.

## Materials and methods

### Data acquisition and analysis

The standardized pan-cancer dataset was downloaded from UCSC (https://xenabrowser.net/): TCGA TARGET GTEx (PANCAN, N=19131, G=60499). A log2(x+1) transformation was applied to each expression value, and cancer types with fewer than 3 samples were excluded, resulting in the final expression data for 34 cancer types. Additionally, prognostic data for TCGA were sourced from prior studies (24). Simultaneously, TARGET follow-up data were supplemented from the UCSC database. Samples with a follow-up time of less than 30 days were excluded, and cancer types with fewer than 10 samples were also excluded. The abbreviations section provides the full names and corresponding abbreviations of the tumors.

The Ph^+^ B-ALL data was downloaded from the GEO database. RMA normalization was performed using the RMA algorithm the NimbleScan 2.5 software. The dataset GSE34861 comprises 191 adult B-ALL samples and 3 normal pre-B samples, and 78 are Ph^+^ B-ALL samples in B-ALL samples.

### Genetic and epigenetic alterations in pan-cancer

Genomic alteration data and methylation data were download from cBioPortal database (https://www.cbioportal.org/). The correlation between *LAPTM4B* expression and gene promoter methylation was evaluated using Spearman rank correlation. Kaplan−Meier analysis was performed to analyze the relationship between *LAPTM4B* methylation and the prognosis of patients.

### Clinical characteristics *LAPTM4B* in pan-cancer

We developed the Cox proportional hazards regression model to analyze overall survival (OS), disease-specific survival (DSS), disease-free interval (DFI), and progression-free interval (PFI) of *LAPTM4B* across cancers. Kaplan−Meier analysis was performed to analyze the prognostic significance.

The diagnostic significance of *LAPTM4B* across cancers was assessed by the Receiver Operator Characteristic (ROC) curve via “pROC” (v1.17.0.1). The diagnosis accuracy was evaluated by the Area under Curve (AUC). The AUC is closer to 1, the diagnosis accuracy is better.

The IC_50_ values of various compounds in cancer cell lines were obtained from the GDSC dataset (https://www.cancerrxgene.org), to assess the relationship between *DLAT* and the drug response of tumor cells by the Spearman correlation coefficient. A higher IC50 indicates that cancers are less sensitive to the compounds.

### Tumor immune microenvironment analysis

Tumor-infiltrating lymphocytes (TILs) participated in predicting sentinel node status and associated with prognosis (25). ssGSEA scores of the correlation between *LAPTM4B* and immune cell infiltration for Ph^+^ B-ALL were calculated using the xCELL algorithm and TILs. Spearman rank correlation was employed to assess the association between *LAPTM4B* expression and immune cell infiltration in pan-cancer, utilizing the xCELL and CIBERSORT algorithms.

Immune checkpoint-related genes (ICGs) were obtained from a previous study (26). Immune-related genes were downloaded from the TISIDB database (http://cis.hku.hk/TISIDB/index.php). The relationship of immune-related genes and *LAPTM4B* in Ph^+^ B-ALL was evaluated using ssGSEA. Immune regulatory genes are distributed in five immune pathways, including chemokine (41 genes), receptor (18 genes), MHC (21 genes), immunoinhibitor (24 genes) and immunostimulator (46 genes). The relationship between immune-related genes and *LAPTM4B* expression in pan-cancer was evaluated by Spearman rank correlation.

### Tumor microenvironment analysis in pan-cancer

We obtained 10,180 tumor samples from a total of 44 tumor types for immune infiltration scores. ESTIMATE was used to reflect the degree of infiltration of stromal or immune cells into tumors. The ESTIMATE algorithm included stromal, immune, and ESTIMATE scores. Spearman rank correlation was used to evaluate the correlation between *LAPTM4B* expression and these three scores by the R software packages “estimate” and “psych”.

We downloaded all level 4 simple nucleotide variation data of TCGA samples from GDC (https://portal.gdc.cancer.gov/). Tumor mutation burden (TMB) was analyzed using MAftools package (Version 2.8.05) of R software. Tumor stem cell infiltration analysis was performed based on DNA methylation dry score (DNAss) and RNA dry score (RNAss) (27). Spearman rank correlation was used to evaluate the correlation between *LAPTM4B* expression and TMB, microsatellite instability (MSI), purity, DNAss and RNAss.

### Single-cell analysis and enrichment analysis

We conducted the single-cell level expression of *LAPTM4B* at in leukemia using TISCH2 (28). TISCH2 encompasses 190 tumor scRNA sequence datasets with 6 million cells across 50 cancer types. To assess the functional and signaling aspects with *LAPTM4B*, we conducted Gene Set Enrichment Analysis (GSEA) on HALLMARK and KEGG pathways. Based on the median expression of *LAPTM4B* in cancer, the group was divided into high and low expression groups.

### The development of *LAPTM4B* knockout Ph^+^ B-ALL model and *in vitro* assay

The B6. *LAPTM4B^loxp/loxp^* mice were generated at Biocytogen Pharmaceuticals (Beijing) Co., Ltd, which were intercrossed with B6.CMV-Cre mice to generate B6. *LAPTM4B* ^-/-^ mice. BCR-ABL induced B-ALL model was developed as previously described (29). Briefly, bone marrow (BM) cells were collected from 8-week-old *WT* and *LAPTM4B^-/-^* mice (n=3) and resuspended with BCR/ABL viral infection medium, centrifugation at 1000g for 90min at 37°C, then cultured at 37°C for 3 h. Then, viral transfected cells were injected into lethally irradicated recipient mice at a dosed of 1x10^5 B cells/mouse via the tail vein.

After the *WT* or *LAPTM4B* ^-/-^ BM cells were transfected with BCR/ABL, then seeded in DMEM medium containing 10% FBS in 24-well plates at series initial cell numbers of 5x10^5 (500k), 3x10^5 (300k), 1x10^5 (100k), 3x10^4 (30k), 1x10^4 (10k), and 2.5x10^3 (2.5k). Each well cell numbers were adjusted to 1x10^6 cells/well with WT mice BM cells and cultured with DMEM medium containing 10% FBS. The cell number in each well was counted on day 7 post seeding.

### Cell cycle experiments by the BrdU incorporation method

BrdU was added directly into the prepared cell medium at final concentration of 10 uM, and incubated for 1h. After collection and washing, cells were suspended with PBS containing 0.5% paraformaldehyde on ice for 20 minutes. Then cells were washed with PBS and resuspended with 70% ethanol overnight. Next day, after washing with PBS, cells were resuspended with 2N HCL/0.5% triton X-100 at room temperature for 20 min to denature. After neutralization with 0.1M sodium borate, cells were suspended with PBS containing 0.5% BSA and 0.5%Tween 20 and stained with anti-BrdU antibody-FitC (BD Biosciences) at room temperature for 20min. The cells were resuspended with PBS containing RNase and incubated at 37 ℃. After adding PI, cells were analyzed using flow cytometry.

### Statistical analysis

R software (version 4.2.1) was utilized for this analysis. The Wilcoxon’s test and analysis of variance (ANOVA) were applied for comparisons involving two and multiple groups, respectively. Spearman correlation coefficient was employed for correlation analysis. All experiments were conducted in triplicate.

## Results

### Expressions and alterations of *LAPTM4B* in human cancers

**Figure 1:**
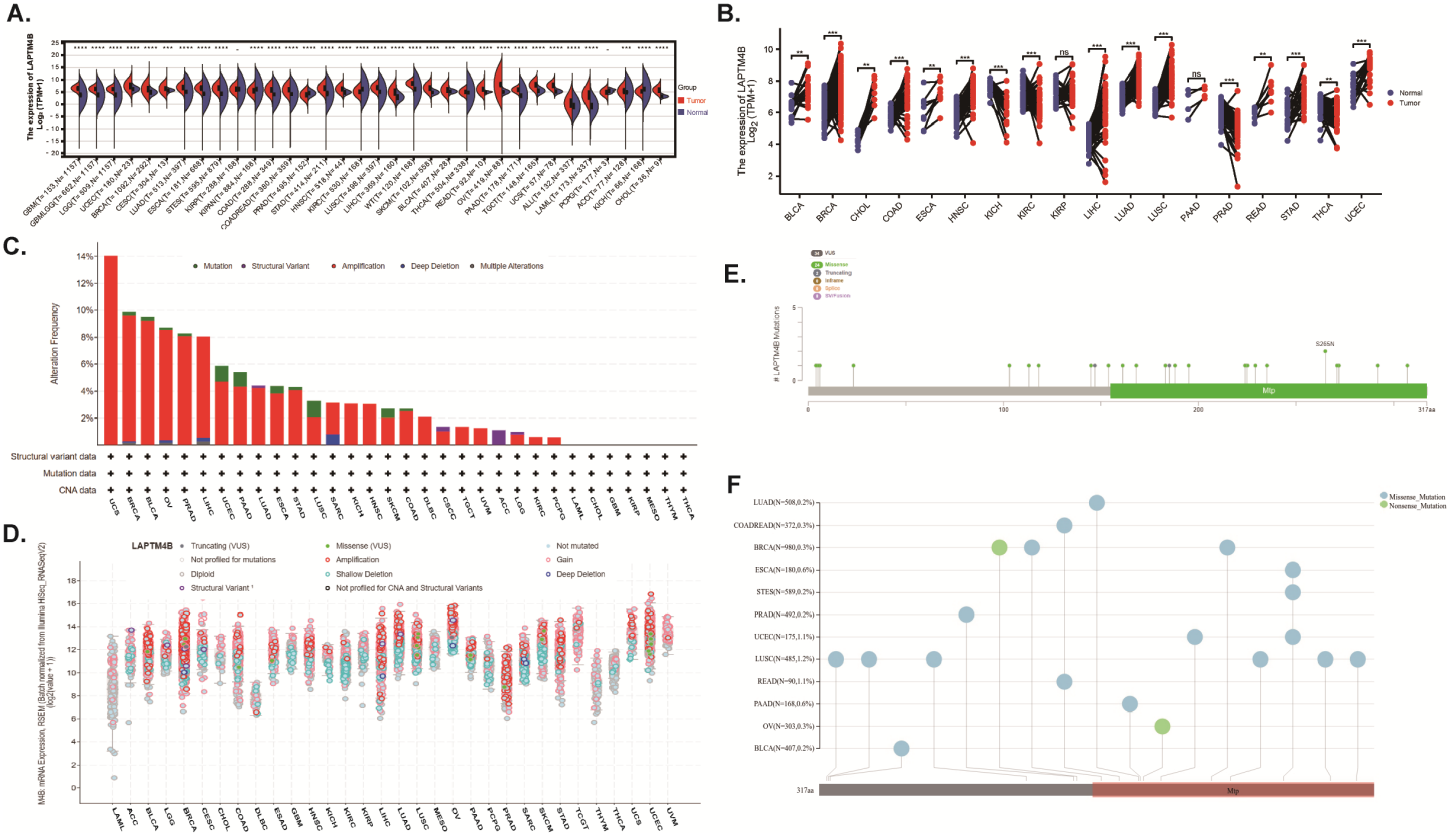
The expression and genetic alteration analysis of *LAPTM4B* across cancers. **(A)** *LAPTM4B* expression levels in tumor and normal samples. **(B)** Paired differential analysis of *LAPTM4B* expression in matched tumor and normal samples from TCGA. **(C)** Bar chart of *LAPTM4B* mutations across cancers. **(D)** Mutation counts and types of *LAPTM4B* across cancers. **(E)** Mutation diagram of *LAPTM4B* across protein domains. **(F)** Landscape of genetic mutation of *LAPTM4B* across cancers. * p<0.05, ** p<0.01, *** p<0.001, **** p<0.0001, ns means non significance.

In order to examine the expression profile of *LAPTM4B* in pan-cancers, we evaluated its expression across 34 cancer types using data from TCGA, TARGET, and GTEx databases. Our findings revealed high *LAPTM4B* expression in 28 cancer types compared to normal tissues, including glioblastoma (GBM), lower-grade glioma (LGG), uterine corpus endometrial carcinoma (UCEC), breast invasive carcinoma (BRCA), cervical squamous cell carcinoma and endocervical adenocarcinoma (CESC), lung adenocarcinoma (LUAD), esophageal carcinoma (ESCA), stomach and esophageal carcinoma (STES), colon adenocarcinoma (COAD), colon adenocarcinoma/Rectum adenocarcinoma esophageal carcinoma (COADREAD), stomach adenocarcinoma (STAD), head and neck squamous cell carcinoma (HNSC), lung squamous cell carcinoma (LUSC), liver hepatocellular carcinoma (LIHC), high-risk Wilms tumor (WT), skin cutaneous melanoma (SKCM), bladder urothelial carcinoma (BLCA), thyroid carcinoma (THCA), rectum adenocarcinoma (READ), ovarian serous cystadenocarcinoma (OV), pancreatic adenocarcinoma (PAAD), testicular germ cell tumors (TGCT), uterine carcinosarcoma (UCS), acute lymphoblastic leukemia (ALL), acute myeloid leukemia (LAML), Adrenocortical carcinoma (ACC), and cholangiocarcinoma (CHOL). While, low *LAPTM4B* expression was observed in 4 cancer types, Pan-kidney cohort (KIPAN), prostate adenocarcinoma (PRAD), kidney renal clear cell carcinoma (KIRC), and kidney chromophobe (KICH) (**Figure 1A**). Specifically, *LAPTM4B* exhibited high expression in BLCA, BRCA, CHOL, COAD, ESCA, HNSC, LIHC, LUAD, LUSC, READ, STAD, and UCEC, while showing low expression in KICH, KIRC, PRAD, and THCA compared to adjacent paired normal tissues (**Figure 1B**). These results suggest that elevated *LAPTM4B* expression is associated with cancer progression in a majority of cases.

The amplification of *LAPTM4B* was observed most frequently in UCS, BRCA, BLCA, OV, PRAD, and LIHC, (**Figure 1C**), and common in most cancers (**Figures 1C, D**). Moreover, we found 34 mutation sites between amino acids 0 and 317, including 24 missense mutations, 2 truncating, 8 SV/fusion, and S265N as the most frequent mutation sites within *LAPTM4B* across cancers (**Figures 1E, F**).

To elucidate potential associations between *LAPTM4B* and intracellular epigenetic alterations, we examined the status of genomic methylation and the expression of genes involved in mRNA methylation in various types of cancer cells using data from cBioPortal database. We found that there were significant negative correlations between *LAPTM4B* expression and gene promotor methylation in most tumors (**Supplementary Figure 1A**). Increased methylation of *LAPTM4B* mRNA was related to poorer OS in patients with GBM and LGG (**Supplementary Figures 1B, C**). Furthermore, the relationships between *LAPTM4B* and genes involved in mRNA m1A, m5C, m6A modifications were evaluated. *LAPTM4B* expression was significantly positively related to these RNA modification genes in almost all tumors (**Supplementary Figure 1D**). These results indicated that *LAPTM4B* could influence tumor development by regulating the repair of RNA and DNA methylation in cancers.

### Treatment outcome associated with *LAPTM4B* alterations in pan-cancers

**Figure 2:**
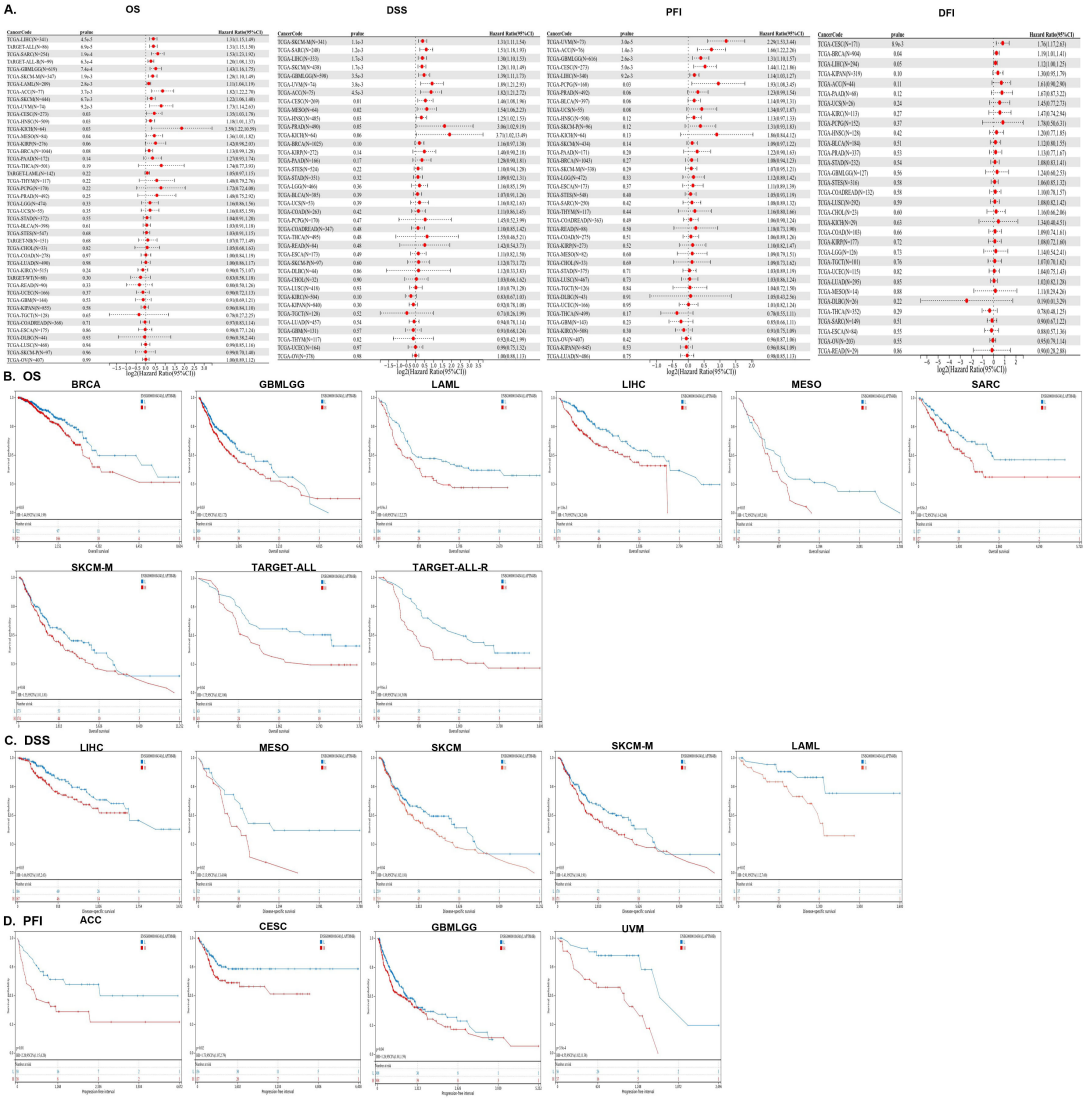
The prognostic analysis of LAMPTM4B across cancers. **(A)** Forest plots of *LAPTM4B* by univariate Cox regression analysis across cancers. OS, DSS, PFI, and DFI. Kaplan−Meier curves showing the relationships of *LAPTM4B* expression with **(B)** OS. **(C)** DSS. **(D)** PFI in pan-cancer.

To evaluate the clinical significance of elevated *LAPTM4B* expression in various cancers, we conducted a Cox proportional hazards model analysis encompassing OS, DSS, DFI, and PFI. Univariate Cox regression analysis of OS, DSS, PFI, and DFI revealed that *LAPTM4B* served as a significant risk factor for patients in multiple cancer types, including LIHC, B-ALL, SARC, GBMLGG, SKCM, AML, ACC, UVM, CESC, HNSC, KICH, MESO, UVM, BRCA, and PCPG (**Figure 2A**). Additionally, Kaplan−Meier survival analyses of OS, DSS, and PFI were further explored across cancers (**Figures 2B–D**).

**Figure 3:**
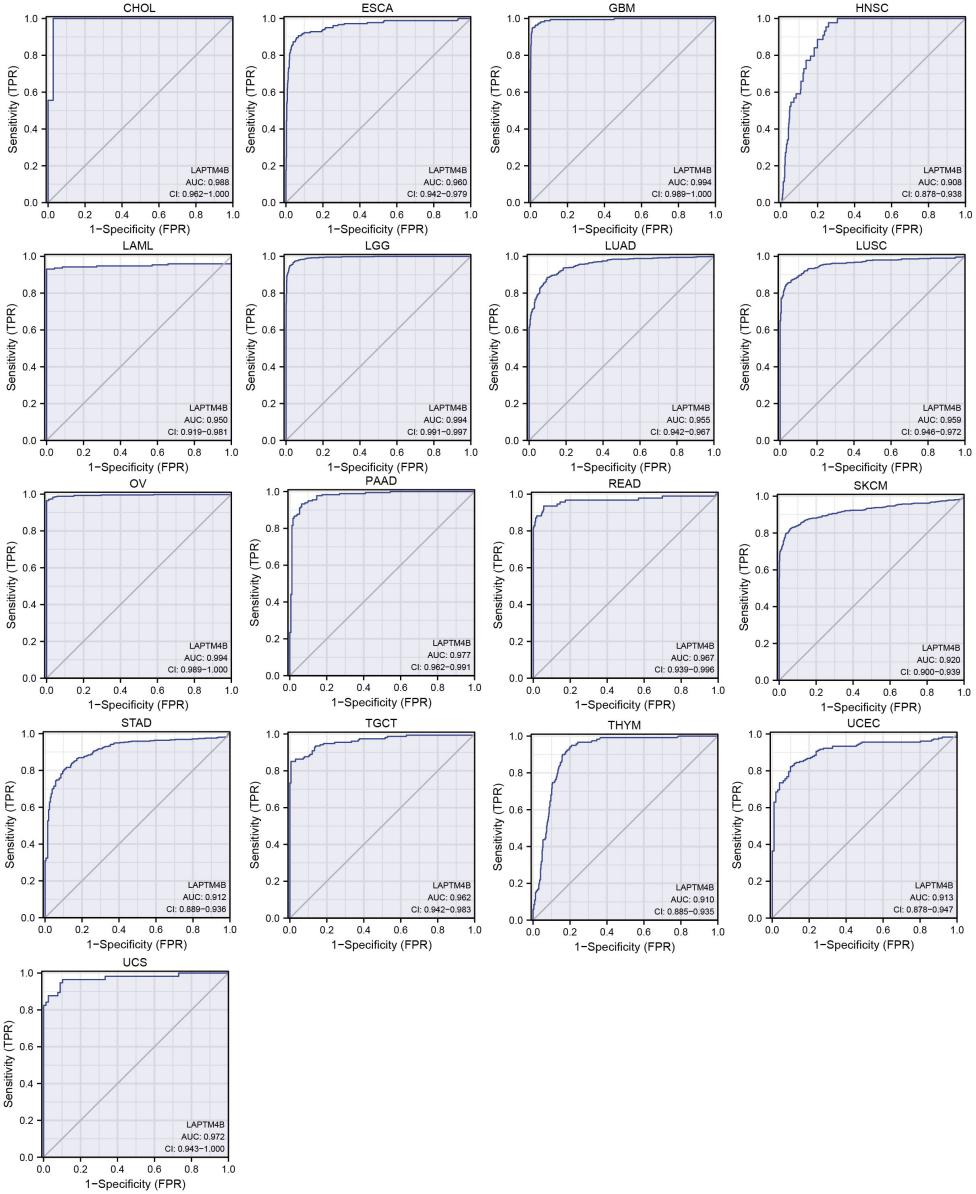
ROC curve of *LAPTM4B* expression in the TCGA and GTEx database in pan-cancer. Cancers with AUC > 0.9.

The performance of the gene signature for diagnostic accuracy was evaluated by the ROC curves. **Figure 3** showed that 17 types of cancer had high diagnostic accuracy (AUC >0.9), including CHOL, ESCA, GBM, HNSC, LAML, LGG, LUAD, LUSC, OV, PAAD, READ, SKCM, STAD, TGCT, THYM, UCEC and UCS. These results suggested that *LAPTM4B* had good diagnostic value in a variety of cancers. The detailed results of all cancers were exhibited in the **Supplementary Table 1**.

To assess the potential correlation between elevated *LAPTM4B* expression and the drug response of tumor cells, we conducted Spearman correlation coefficient analysis using data from the GDSC dataset. Our findings revealed increased *LAPTM4B* expression had increased IC50 values of 14 compounds, including rTRAIL, B-Raf inhibitors (PLX-4720, dabrafenib, SB590885), FTI-277 (FTase inhibitor), bexarotene (RXR agonist), dactolisib (PI3K/mTOR inhibitor), luminespib (HSP90 inhibitor), palbociclib (CDK4/6 inhibitor), (5Z)-7-Oxozeaenol (TAK1 inhibitor), QS11 (ARFGAP1 inhibitor), among others, which suggested that increased *LAPTM4B* lead drug resistance. Conversely, a negative association was observed with elesclomol and afatinib (EGFR/HER2 inhibitor) responses (**Table 1**). These results suggest that increased *LAPTM4B* expression may confer resistance to a broad spectrum of therapeutic agents in tumor cells. Moreover, we also found that *LAPTM4B* was positively correlated with RNAss and DNAss across most of the cancers (**Supplementary Figures 2A, B**), which indicates that high expression of *LAPTM4B* might be associated with cancer tumor recurrence and metastasis.

**Table 1:** Summary of Spearman’s correlation between *LAPTM4B* expression and drug response (IC50 value) in cancer cell lines based upon the GDSC dataset.

Compound	Correlation	P
PLX-4720	0.124416709	0.00090156
Dabrafenib	0.11962461	0.001516679
FTI-277	0.114908293	0.007683379
Bexarotene	0.113597156	0.009879376
Dactolisib	0.111929208	0.005476148
SB590885	0.110085002	0.004245134
Luminespib	0.107308826	0.011816945
Palbociclib	0.102470636	0.012099797
BX795	0.10112307	0.008624826
(5Z)-7-Oxozeaenol	0.091295862	0.019248295
rTRAIL	0.086089865	0.033004286
GSK269962A	0.084235228	0.031895828
NSC-87877	0.080602051	0.038372314
QS11	0.077092171	0.044075024
Elesclomol	-0.081062898	0.049755553
Afatinib	-0.102577808	0.006747785

### Immune status analysis of *LAPTM4B* in pan-cancer

**Figure 4:**
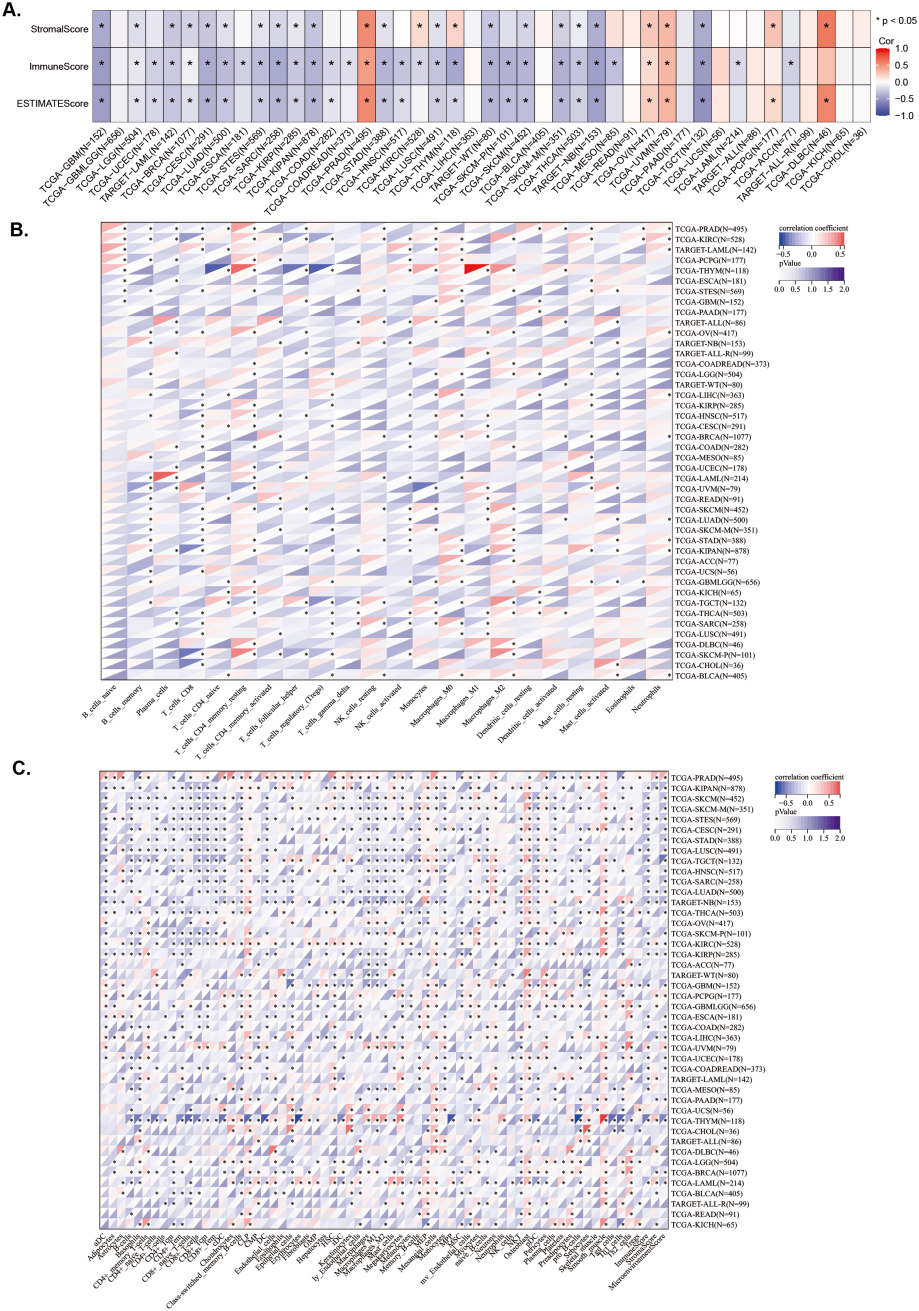
Association between LAPTM4B expression and immune status across cancers. **(A)** Relationship between LAPTM4B expression and the StromalScore, ImmuneScore, and ESTIMATEScore. Relationships between LAPTM4B expression and the immune cells by CIBERSORT algorithm **(B)**, and the xCell algorithm **(C)**. * p<0.05, ** p<0.01, *** p<0.001, **** p<0.0001, ns means non significance.

To explore the relationship between *LAPTM4B* expression and immune status in pan-cancer, we conducted a correlation analysis. Overall, we found that *LAPTM4B* expression was associated with immune subtypes in 19 cancer types and correlated with molecular subtypes in 14 cancer types (**Supplementary Figures 3A, B**). Additionally, we analyzed stromal and immune cell scores to investigate the relationship between *LAPTM4B* expression and the tumor immune microenvironment (TIME) across cancers. We observed a positive correlation between *LAPTM4B* expression and StromalScore, ImmuneScore, and ESTIMATEScore in PAAD, OV, and UVM (**Figure 4A**). While, *LAPTM4B* expression showed a negative correlation with these scores in GBM, LGG, LAML, BRCA, CESC, LUAD, STES, SARC, KIRP, KIPAN, STAD, LUSC, WT, SKCM, SKCM-M, THCA, NB, and TCGT (**Figure 4A**). To explore the correlation between *LAPTM4B* expression and immune cells, we developed a heat map of *LAPTM4B* with immune cells by CIBERSORT and xCell. Our result revealed that *LAPTM4B* was associated with CD8^+^ T cells, macrophages M2 and Tregs in many cancers, which suggested that high *LAPTM4B* expression had inhibitory immune microenvironment (**Figures 4B, C**). Overall, our findings suggested that elevated *LAPTM4B* expression might be associated with a potential decrease in patients’ immune anti-tumor capabilities.

**Figure 5:**
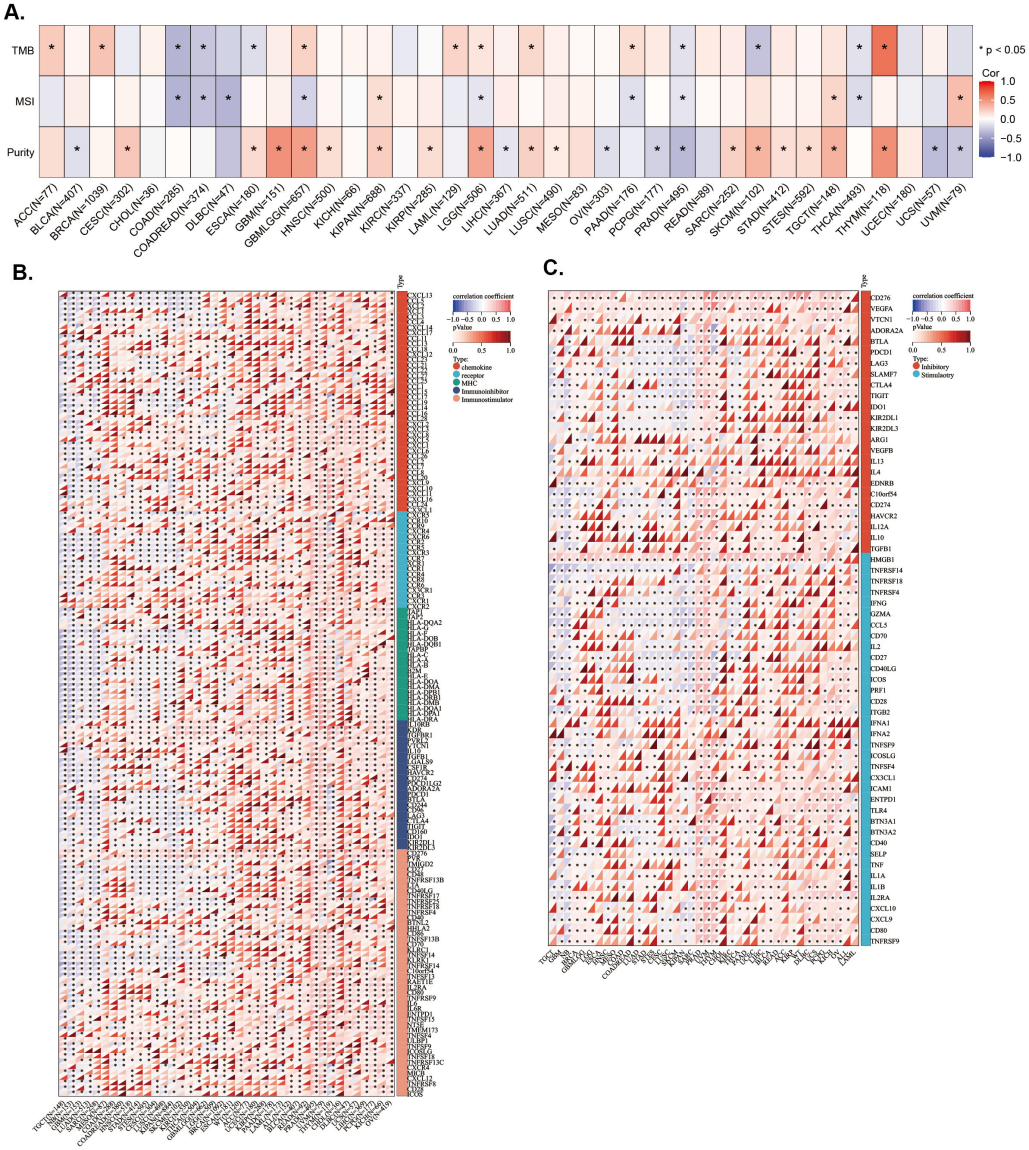
Association between *LAPTM4B* expression and genes in microenvironment immune cells across cancers. Relationship between *LAPTM4B* and TBM, MSI, and purity. Correlation between *LAPTM4B* and immune regulatory genes **(A)**, and immune checkpoint genes **(B, C)**. *p<0.05.

To investigate whether *LAPTM4B* expression levels are associated with TMB, MSI, and tumor purity, we conducted analyses using Spearman correlation analysis. The results showed that *LAPTM4B* expression was positively correlated with TMB in ACC, BRCA, GBMLGG, LAML, LGG, LUAD, PAAD, and THYM, while exhibiting a negative correlation in COAD, COADREAD, ESCA, PRAD, SKCM, and THCA (**Figure 5A**). The MSI analysis revealed a positive correlation of *LAPTM4B* expression with MSI in KIPAN, TGCT, and UVM, while a negative correlation in COAD, COADREAD, DLBC, GBMLGG, LGG, PAAD, PRAD, and THCA (**Figure 5A**). Additionally, *LAPTM4B* showed a significant correlation with tumor purity, with positive associations in CESC, ESCA, GBM, GBMLGG, HNSC, KIPAN, KIRP, LGG, LUAD, LUSC, SARC, SKCM, STAD, STES, TGCT, and THYM, and negative associations in BLCA, LIHC, OV, PCPG, PRAD, UCS, and UVM (**Figures 5A**). These findings suggest that *LAPTM4B* expression might serve as a potential biomarker for immunotherapy.

Subsequently, the correlations of expression levels between *LAPTM4B* and immune checkpoint genes and immune regulatory genes in cancers were also investigated. We found that *LAPTM4B* expression was positively related to immune regulatory genes in majority tumor types, especially in PRAD, UVM, THYM, LIHC, BLCA, and OV. While, *LAPTM4B* expression was negatively related to immune regulatory genes in TGCT, GBM, LUAD, SARC, KIPAN, and SKCM (**Figure 5B**). Additionally, *LAPTM4B* expression was positively related to immune checkpoint genes in most types of tumors, except for some tumors, which were mainly TGCT, GBM, SKCM, and SARC (**Figure 5C**). In general, these results suggested that *LAPTM4B* might regulate immune cell infiltration and immune-related genes functions in most tumor types.

### Single-cell and enrichment analysis of *LAPTM4B* expression in leukemia

**Figure 6:**
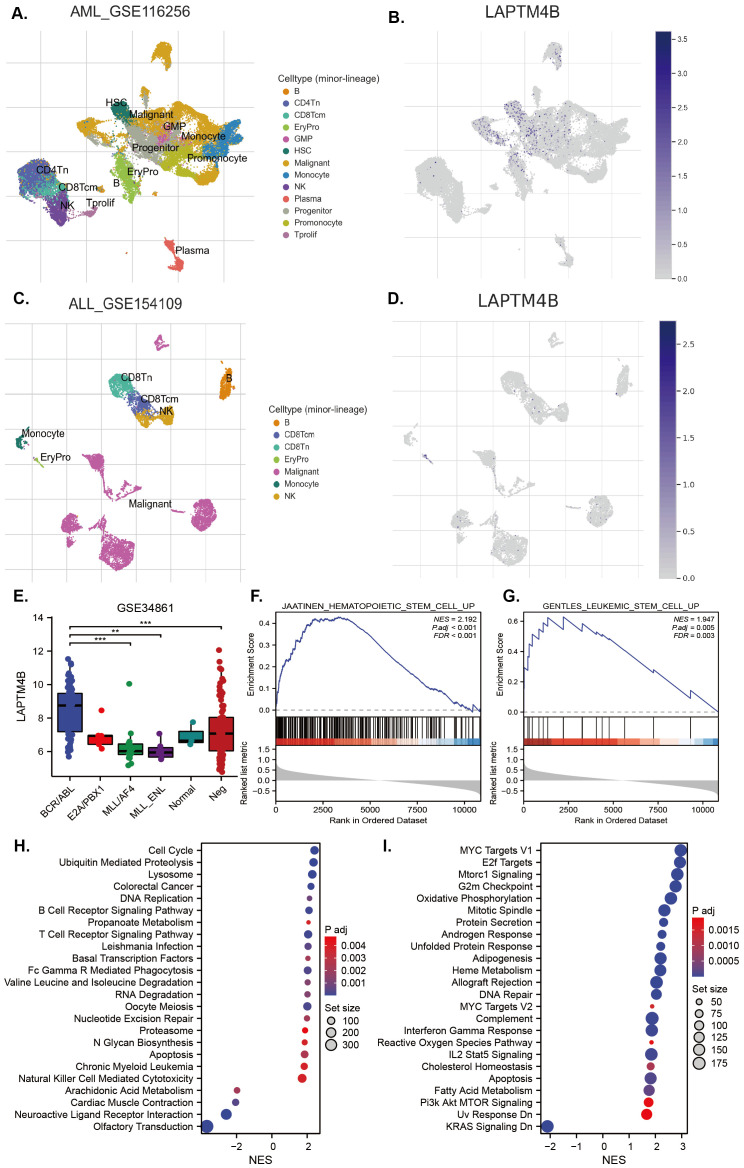
Expression of *LAPTM4B* at the single-cell level and its related signaling in B-ALL. *LAPTM4B* expression profiles at single-cell level in AML **(A, B)**, and B-ALL **(C, D)**. The expression of *LAPTM4B* in different subtypes in B-ALL **(E)**. GSEA analysis of *LAPTM4B* was related to hematopoietic stem cells **(F)**, and leukemia stem cells **(G)** in Ph+ B-ALL. KEGG **(H)** and HALLMARK **(I)** analysis suggested that *LAPTM4B* was correlated with the cell cycle, MYC, E2F, and G2M checkpoint pathways in Ph^+^ B-ALL. * p<0.05, ** p<0.01, *** p<0.001, **** p<0.0001, ns means non significance.

*LAPTM4B* could be a diagnostic, prognostic or therapeutic factor in hepatocellular carcinoma, breast cancer, bladder cancer, renal clear cell carcinoma, nasopharyngeal cancer, lung cancer, osteosarcoma, glioblastoma, gastric cancer, pancreatic ductal adenocarcinoma, ovarian cancer, neck squamous cell carcinomas, prostate cancer, endometrial cancer, colorectal cancer, gallbladder carcinoma, esophageal cancers, cervical carcinoma, melanoma, pancreatic carcinoma and AML and so on (17, 18, 20, 30–51). However, none study was performed in ALL, so we focused on ALL, particularly Ph^+^ B-ALL, to elucidate and clarify the biological functional characteristics of *LAPTM4B*. Taking the advantages of single-cell sequencing and open public data, we found that *LAPTM4B* was expressed mainly in normal HSCs, progenitors, and AML cells (**Figures 6A, B**). In an ALL sample, we found that *LAPTM4B* was highly expressed in proerythroblasts, but not malignant cells (**Figures 6C, D**). Interesting, an analysis based on an expression profiling of 191 B-ALL samples and 3 normal pre-B samples showed that *LAPTM4B* was more highly expressed in BCR/ABL B-ALL than other subtypes (**Figure 6E**). Then, we performed the analysis of the function and pathways of *LAPTM4B*-related genes in Ph^+^ B-ALL. We found that genes associated with HSCs and leukemia stem cells (LSCs) were up-enriched in high *LAPTM4B* expression samples (**Figures 6F, G**), as well as genes associated with cell cycle, DNA replication, MYC target, E2F and G2M checkpoint pathways were also up-enriched in in Ph^+^ B-ALL (**Figures 6H–I**).

### Relationships between *LAPTM4B* expression and immune status in Ph^+^ B-ALL

**Figure 7:**
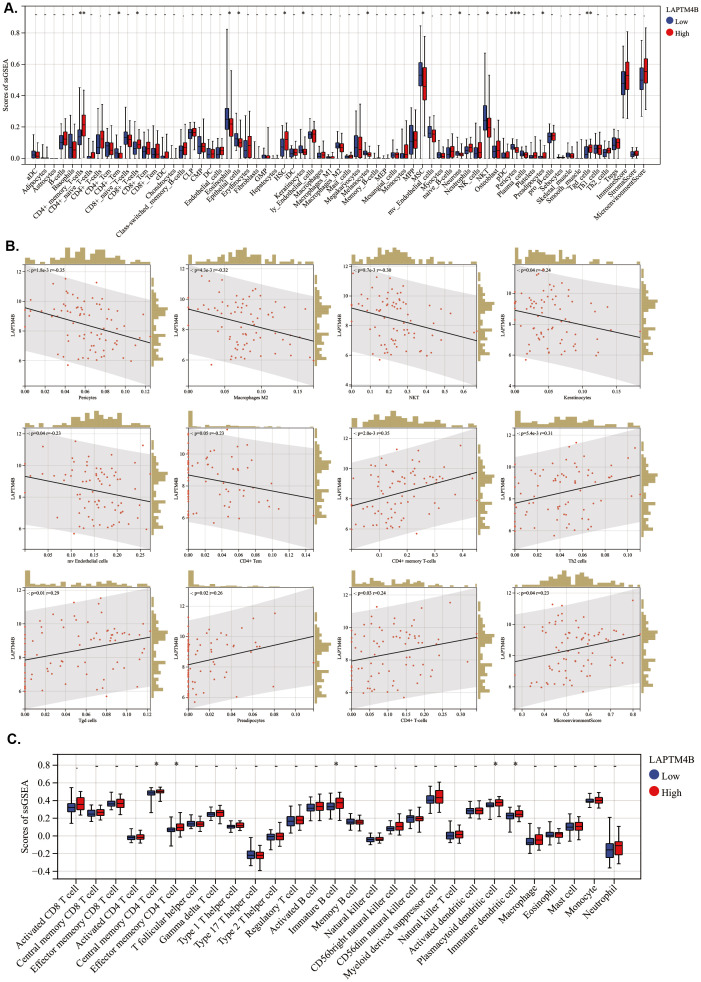
Association between *LAPTM4B* expression and immune-related cells in ph+ B-ALL. **(A)** Boxplots of immune cells in different *LAPTM4B* expression groups by xCell algorithm. **(B)** Scatterplot of *LAPTM4B* correlated with immune cells. **(C)** Boxplots of TILs in different *LAPTM4B* expression groups. * p<0.05, ** p<0.01, *** p<0.001, **** p<0.0001, ns means non significance.

To evaluate the association of *LAPTM4B* expression with TME in Ph^+^ B-ALL, we conducted an ESTIMATE analysis to calculate the stromal score, immune score, ESTIMATE score, and tumor purity within Ph^+^ B-ALL. We found that *LAPTM4B* expression was not significantly associated with TME in Ph^+^ B-ALL (**Supplementary Figure 4**). Then, the relationship between *LAPTM4B* and immune cells in Ph^+^ B-ALL was conducted using xCell algorithm method. The scores of CD4^+^ memory T cells, CD8^+^ T cells, HSC, preadipocytes, and Tgd cells were higher; while, the scores of CD4^+^ Tem, eosinophils, epithelial cells, MSC, and NKT were significantly lower in the high *LAPTM4B* expression patient samples (**Figure 7A**). *LAPTM4B* expression was negatively related to macrophages M2, NKT, mv endothelial cells, and CD4^+^ Tem; while they were positively correlated with CD4^+^ memory T cells, Th2 cells, Tgd cells, CD4^+^ T cells and microenvironment Score (**Figure 7B**). Moreover, immune infiltration scores of tumor-infiltrating lymphocytes (TILs) type in different *LAPTM4B* expression groups were also evaluated using ssGSEA. The central memory CD4 T cells, effector memory CD4 T cells, immature B cells, plasmacytoid dendritic cells, and immature dendritic cells were highly expressed in the high *LAPTM4B* expression patient samples (**Figure 7C**).

The correlation between *LAPTM4B* expression and immune-related genes was also assessed. As previous reports, there were 79 genes related to immune checkpoint (26). We found that *TNFRSF14* and *TNFSF14* were lowly expressed in the high *LAPTM4B* expression samples (**Supplementary Figure 5A**). Additionally, *TNFRSF14* was negatively correlated with *LAPTM4B* expression; whereas, *CTLA4*, *HLA-E*, and *ICOS* were positively associated with *LAPTM4B* expression (**Supplementary Figure 5B**). Moreover, the correlation between *LAPTM4B* and the chemokine genes was also evaluated. We found that *CCL1*, *CCL11*, *CCL15*, *CCL19*, *CCL21*, *CCL22*, *CCL24* and *CCL25* were lowly expressed in high *LAPTM4B* expression samples (**Supplementary Figure 5C**). But, no significant difference was observed on immunoinhibitory genes, immunostimulatory genes, receptor genes, and MHC genes except for *IL6*, *LTA*, *ULBP1*, and *XCR1* (**Supplementary Figures 6A-D**). These results suggested that the high expression of *LAPTM4B* might affected immune microenvironment in Ph^+^ B-ALL.

### *LAPTM4B* deletion impairs the development and progression of Ph^+^ B-ALL

**Figure 8:**
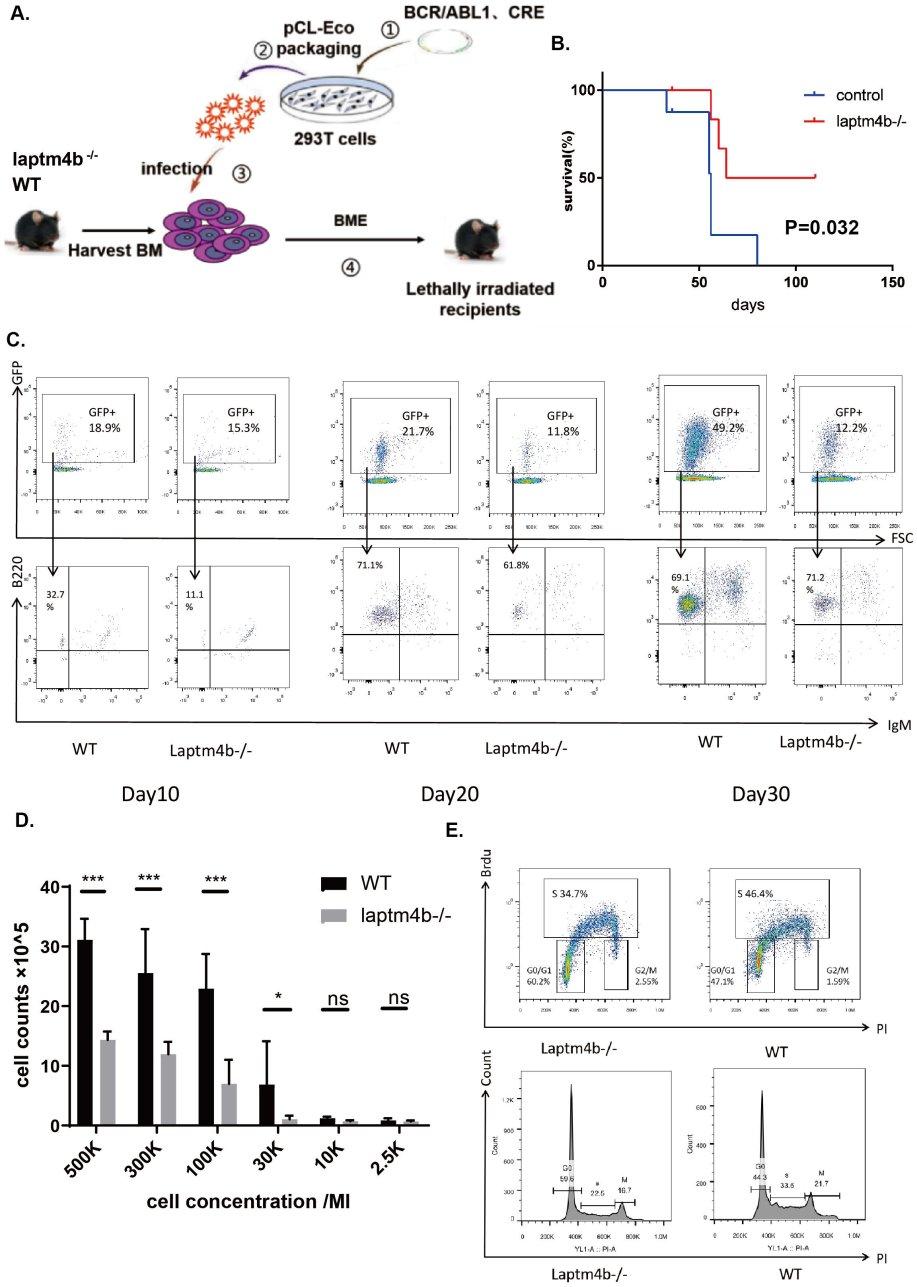
*LAPTM4B* promoted the development and progression of Ph^+^ B-ALL. **(A)** The schematic diagram to establish the mouse model. The Kaplan-Meier curve **(B)**, and the counts of GFP^+^/B22^+^/IgM^-^ Ph^+^ B-ALL cells **(C)** in *LAPTM4B* ^-/-^ and wild type Ph^+^ B-ALL mouse model. The cell counting **(D)**, and the cell cycle **(E)** in *LAPTM4B* ^-/-^ and wild-type Ph^+^ B-ALL cells *in vitro*. * p<0.05, ** p<0.01, *** p<0.001, **** p<0.0001, ns means non significance.

To instigate the involvement of *LAPTM4B* in the development of Ph^+^ B-ALL, we employed a Ph^+^ B-ALL mouse model. Bone marrow (BM) cells from wild type (*WT*) or *LAPTM4B^-/-^* mice were transfected with retrovirus containing BCR/ABL and then injected into lethally irradicated recipients (**Figure 8A**). Overall, the survival time of recipients receiving *LAPTM4B^-/-^* BM cells was significantly longer than that receiving *WT* BM cells (**Figure 8B**). We also monitored the number of leukemic cells with BCR/ABL (represented with GFP and B220) in peripheral blood of mice receiving BCR/ABL-transduced WT or *LAPTM4B^-/-^* BM cells on the day 10, 20 and 30 post-BM transplantation. We found that the percentages of B-lymphoid leukemic cells were significantly lower in mice receiving BCR/ABL-transduced WT or *LAPTM4B^-/-^* BM cells than in those receiving BCR/ABL-transduced *WT* BM cells at all time points measured (**Figure 8C**). To investigate the role of *LAPTM4B* in BCR/ABL-induced leukemogenesis, we conducted an *in vitro* assay for proliferation of BCR/ABL transformed BM B-lymphoid progenitors, as described in methods. BCR-ABL-transformed B-lymphoid progenitors from *LAPTM4B^-/-^* BM cells exhibited much lower number than it transformed those from WT BM cells (**Figure 8D**). Further, *in vitro* Brdu assays for cell proliferation rate showed that *LAPTM4B* deletion impaired Ph^+^ B-ALL cell proliferation and caused G0/G1 arrest (**Figure 8E**). These findings demonstrated that *LAPTM4B* deletion significantly impaired the development and progression of Ph^+^ B-ALL.

## Discussion

*LAPTM4B* is required for lysosomes function, participates in the cell death program, promotes autophagy and tolerance to metabolic stress in cancer cells (52) (53), and is an essential gene for adjuvant drug resistance (15, 54). Our results revealed the role *LAPTM4B* plays in pan-cancer and Ph^+^ B-ALL. We determined the expression levels of *LATPM4B* mRNA in various cancers, and confirmed the most common types of *LAPTM4B* mutations and their locations. The correlation between epigenetic alterations and *LAPTM4B* were evaluated. We also assessed the diagnostic, prognostic and therapeutic values of *LAPTM4B* expression across cancers and differentiated its expression levels across several immune and cellular subtypes of cancers. We associated *LAPTM4B* expression levels with tumor microenvironment and the infiltration levels of immune cells and genes in various cancers. Besides, the expression of *LAPTM4B* at the single-cell level and function in B-ALL were explored. We identified that the loss of the *LAPTM4B* gene impeded BCR-ABL-induced B-ALL progression, both *in vitro* and *in vivo*. We also explored the immunological function of *LAPTM4B* in Ph^+^ B-ALL.

*LAPTM4B* was not highly expressed in all tissues, its expression was high in the testis, heart, skeletal muscle and uterus (11), while we found that it was highly expressed in most cancers. And the amplification was the most frequent alteration. However, the expression of *LAPTM4B* was discovered lowly expressed in KIPAN, KIRC, KICH and PRAD, while the genetic alterations were mainly amplification in these cancers. Therefore, there were other factors affected the expression of *LAPTM4B*. As we known, abnormal methylation of DNA and RNA promotes various diseases and cancers (55–61). And we found that *LAPTM4B* expression was significantly related to DNA methylation and RNA modifications in these cancers. Theses might partly explain the inconsistencies between alteration and expression.

A total of 17 types of cancer had high diagnostic accuracy (AUC >0.9), suggesting that *LAPTM4B* had good diagnostic value. Meanwhile, *LAPTM4B* was a risk factor in many cancers. Besides, we found that increased *LAPTM4B* expression led resistance to a broad spectrum of therapeutic agents in tumor cells. These results were consistent with previous studies that *LAPTM4B* could be a diagnostic, prognostic and therapeutic factor in hepatocellular carcinoma, breast cancer, hepatocellular carcinoma, bladder cancer and renal cell carcinoma and so on (17, 18, 30–38). *LAPTM4B* expression was different in different molecular or immune subtypes of cancer, which results in different survival in the overall population and particular subtype of cancer. Therefore, immune features should be also considered in the further study.

TMB can reflect the proportion of somatic mutations in tumors (62). MSI refers to the arbitrary length change of microsatellites in tumor tissue due to insertion or deletion of repeat units (63). The purity of the tumor usually related to prognosis (64). TMB, MSI, and tumor purity are emerging biomarkers associated with the immunotherapy response. ESTIMATE reflects the degree of infiltration of stromal or immune cells into tumors. High stemness scores represent the activity of tumor stem cells, and are associated with drug resistance and the continuous proliferation of tumor cells, and are correlated with poorer survival (27). Our results exhibited that *LAPTM4B* was significant correlated with these indexes. Besides, we showed that *LAPTM4B* was related to different immune cells in various cancers. In general, the infiltration of activated CD8^+^ T cells, Tem and Tcm CD8+ cells, and Tem CD4+ cells was associated with good prognosis, whereas MDSCs and Tregs were correlated with bad prognosis (65). A previous study identified that *LAPTM4B* inhibited human regulatory T cells produced TGF-β1 (66). Besides, *LAPTM4B* was upregulated in CML TKI-resistant patients (23). Therefore, *LAPTM4B* might be an immunotherapeutic factor in various cancers.

Then, we found that *LAPTM4B* is expressed primarily in stem cells at single-cell level in leukemia, and highly expressed in BCR/ABL subtype of B-ALL, and upregulated stem cell pathway in Ph^+^ B-ALL. A previous study also identified *LAPTM4B* as a candidate gene related to stemness, which was the downstream target of HOXB4 in hematopoietic progenitor cells (67). Similarly, the study verified that *LAPTM4B* was closely related to the stemness of HCC (16). These studies illustrated that *LAPTM4B* might regulate stem cell-related genes. Additionally, *LAPTM4B* might participated in the signaling pathways of MYC, E2F, cell cycle, and T/B cell receptor. And in the Ph+ B-ALL mouse model, *LAPTM4B* knockout prolonged survival, inhibited cell proliferation and arrest of G0/G1. The results were similar to the previous study, which exhibited that *LAPTM4B* promoted the entry of cells from the G1 into the S phase in breast cancer (68).

In B-cell malignancies, leukemic cells can alter the normal microenvironment, in favor of their growth, survival and resistance to cytotoxic therapies (69). Immune cells are important constituents of the tumor stroma and play a crucial role in tumor development and progression60 (70, 71). Our study showed that *LAPTM4B* expression was associated with HSC, preadipocytes, immature B cells, and immature dendritic cells in Ph+ B-ALL. These results validated that *LAPTM4B* was related to stem cell pathways as before analyzed. Moreover, the results demonstrated that high *LAPTM4B* expression had low *TNFRSF14* and *TNFSF14* expression, and was positively correlated with the *CTLA4* and *ICOS* checkpoint gene. *TNFSF14* was an immune-activating gene, mainly expressed in activated T cells, activated natural killer cells and immature dendritic cells (72). TNFRSF14 is a receptor for BTLA, TNFSF14/LIGHT, and homotrimeric TNFSF1, is involved in lymphocyte activation. CTLA4 also known as CD152, is a protein receptor that acts as an immune checkpoint and negatively regulates the immune response (73). ICOS, also called CD278, ICOS were immune checkpoint proteins expressed on activated T cells. Tumor-infiltrating Tregs expressed high levels of cell surface molecules associated with T-cell activation, such as CTLA4, PD-1, LAG3, TIGIT, ICOS, and TNF receptor superfamily members (74). These results suggested that *LAPTM4B* might influence the efficacy of immunotherapy.

In summary, our study systematically performed a comprehensive pan-cancer analysis of *LAPTM4B*, and explored the expression, immunological features, and functions of the *LAPTM4B* gene in the Ph^+^ B-ALL mouse model. We demonstrated the abnormal expression profiles of *LAPTM4B* and it was related to clinical diagnosis, prognosis, genetic and epigenetic alterations, immunological features, and drug response. Additionally, we identified that increased *LAPTM4B* expression was associated with an unfavorable prognosis and promoted the development and progression in Ph^+^ B-ALL, and was related to immune status. These results could help illustrate the underlying oncogenic role and immunological function of *LAPTM4B* in cancers.

## Data Availability

The original contributions presented in the study are included in the article/**Supplementary Material**. Further inquiries can be directed to the corresponding authors.
