# Supplementary material for: A comprehensive prognostic and immune analysis of LAPTM4B in pan-cancer and Philadelphia chromosome-positive acute lymphoblastic leukemia
Source: Front Immunol. 2025 Feb 28;16:1522293. doi: 10.3389/fimmu.2025.1522293 (PMC11906416; doi:10.3389/fimmu.2025.1522293)
Supplement: Supplementary file 1 [file DataSheet1.docx]

**Supplementary materials**


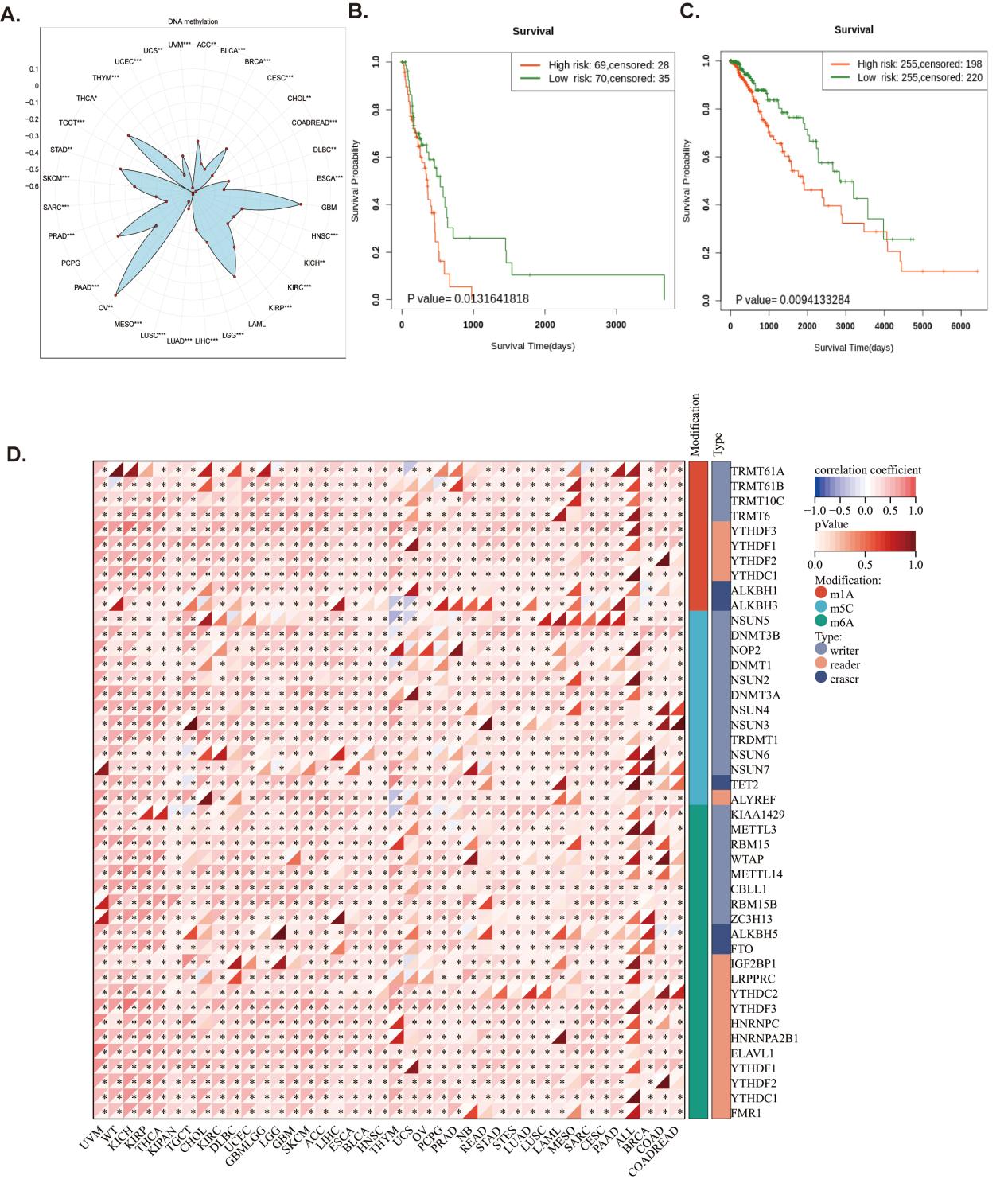


**Supplementary Figure 1. Epigenetic Alterations of LAPTM4B in pan-cancer.** (A) Radar plot showing the correlation between LAPTM4B and promotor methylation. (B) Kaplan‒Meier curves exhibiting the correlations of LAPTM4B promoter methylation levels and OS in GBM. (C) Kaplan‒Meier curves exhibiting the correlations of LAPTM4B promoter methylation levels and OS in LGG. (D) Correlation between LAPTM4B and RNA-modified genes.


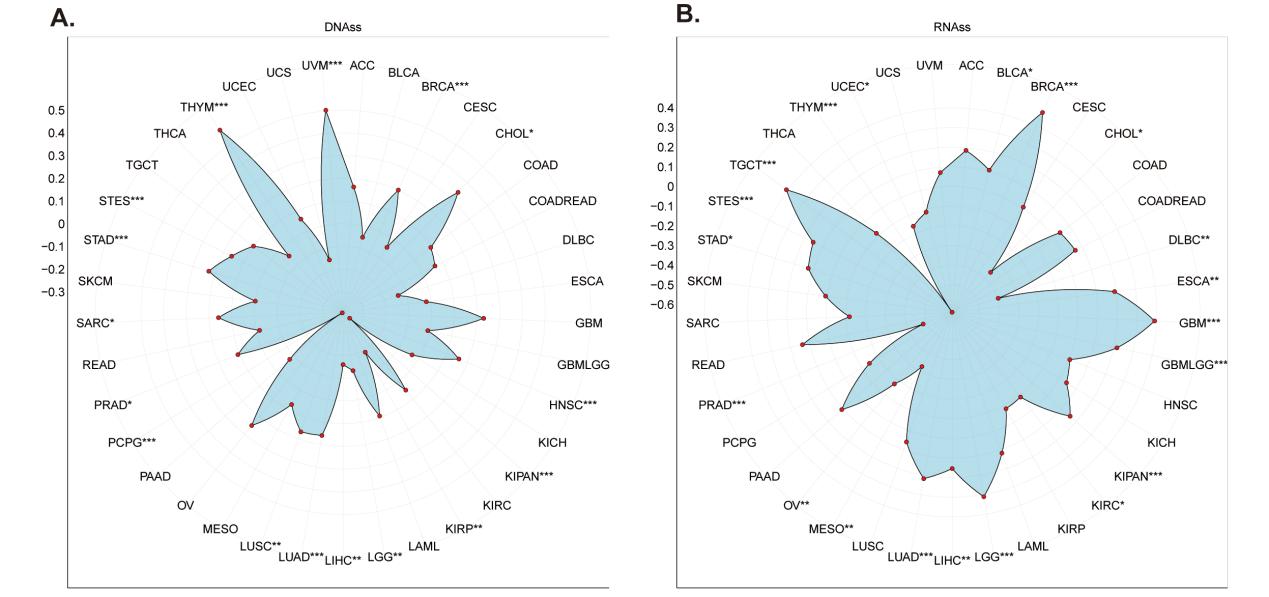


**Supplementary Figure 2.** **Association between LAPTM4B expression and stenmness across cancers**. The radar map showed the relatioships between LAPTM4B expression and DNAss (A) and RNAss (B), the correlation coefficient went from negative number to positive number from the inside to out.


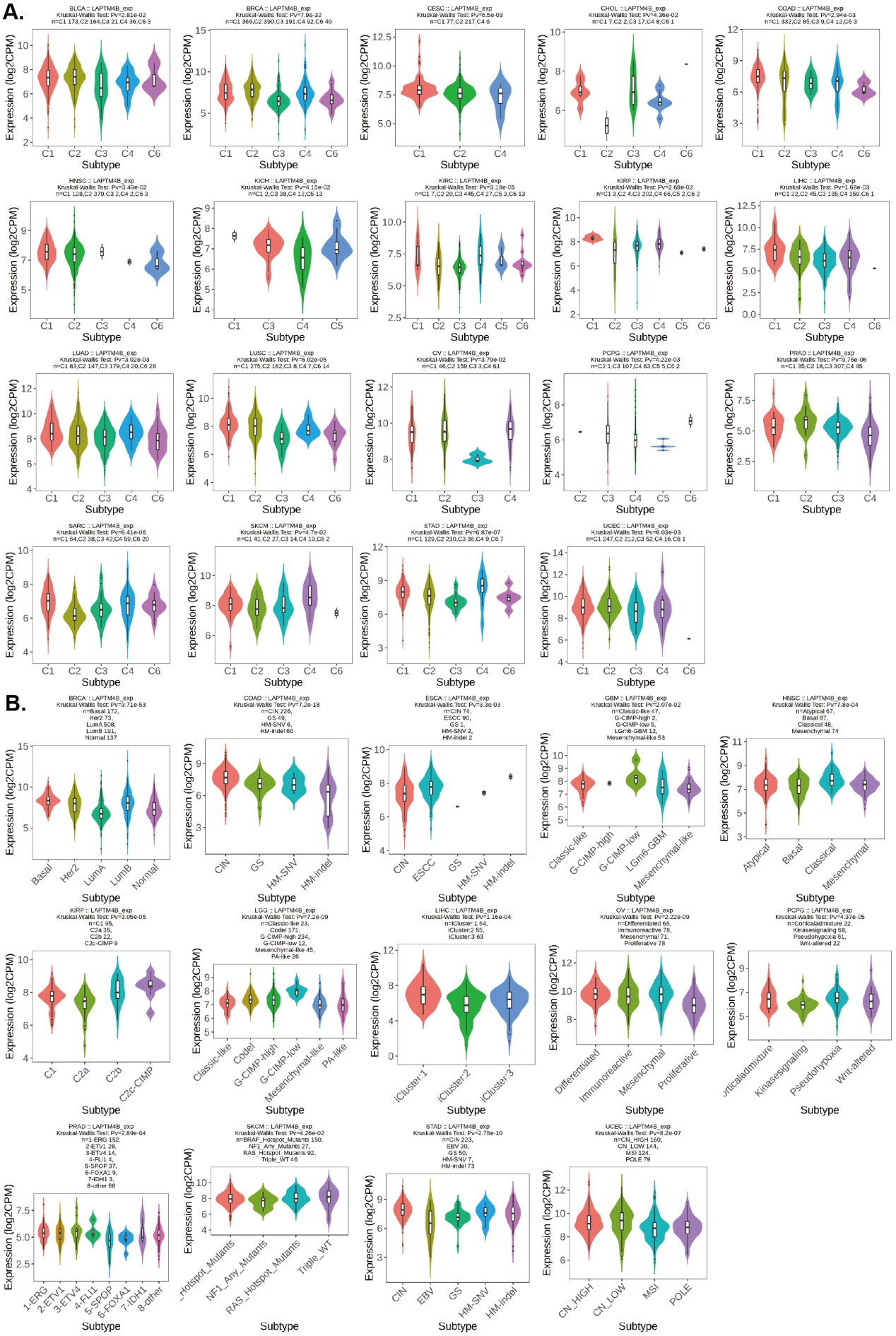


**Supplementary Figure 3.** Correlations between LAPTM4B expression and immune subtypes (A) and molecular subtypes (B) in pan-cacner.


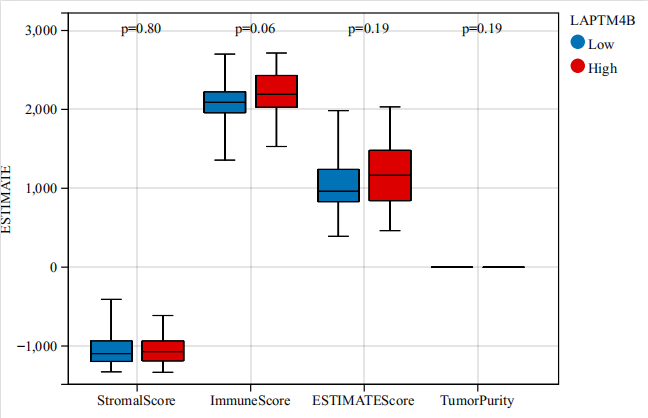


**Supplementary Figure 4. The relationship between LAPTM4B expression and TME in ph+ B-ALL.**


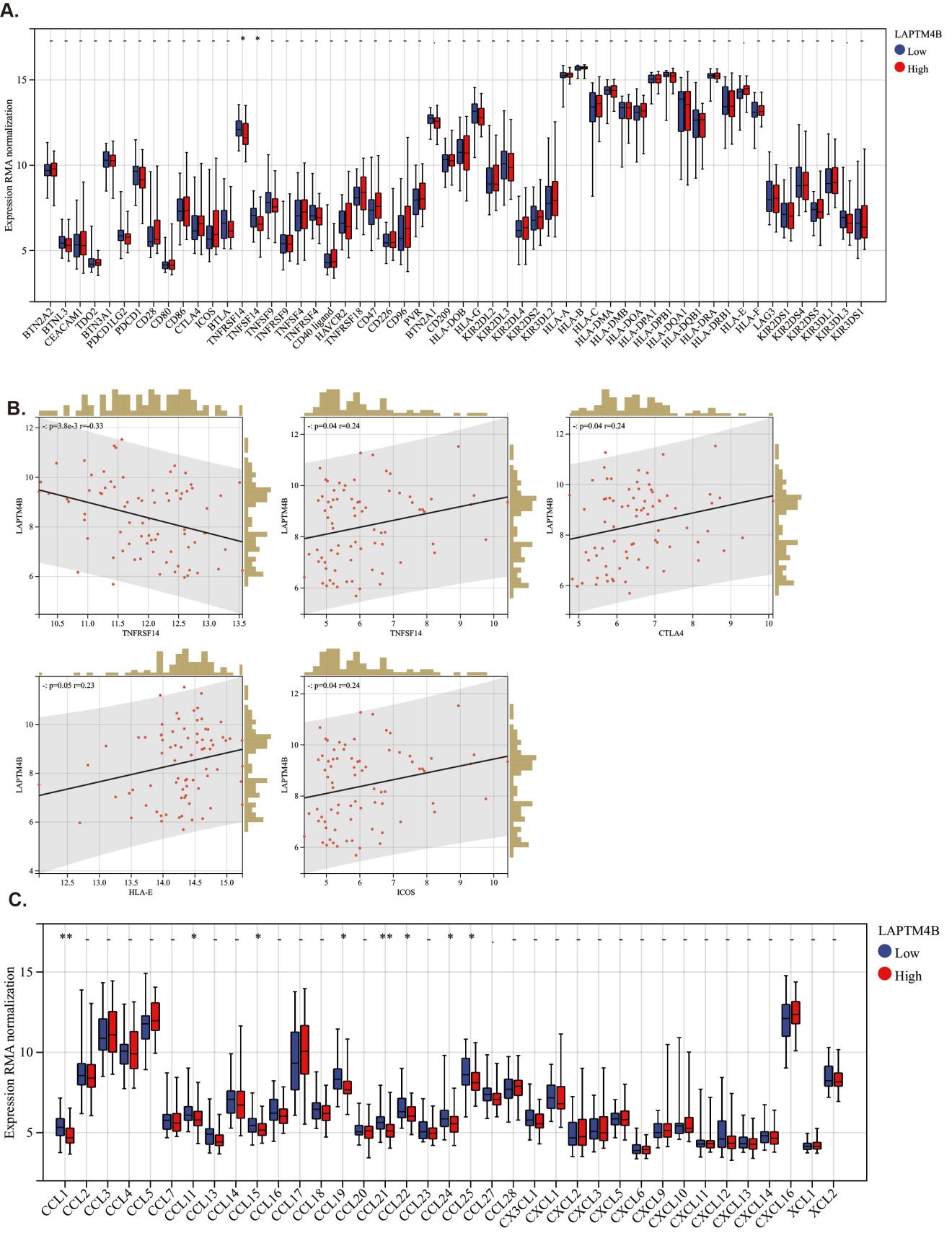


**Supplementary Figure 5.** **Association between *LAPTM4B* expression and immune-related genes in ph+ B-ALL.** (A) Boxplots of immune checkpoint genes in different *LAPTM4B* expression groups. The y axis meaned the scores of ssGSEA. (B) Scatterplot of *LAPTM4B* correlated with immune checkpoint genes. The y axis meaned the *LAPTM4B* expression. (C) Boxplots of chemokine genes in different *LAPTM4B* expression groups. The y axis meaned the scores of ssGSEA.


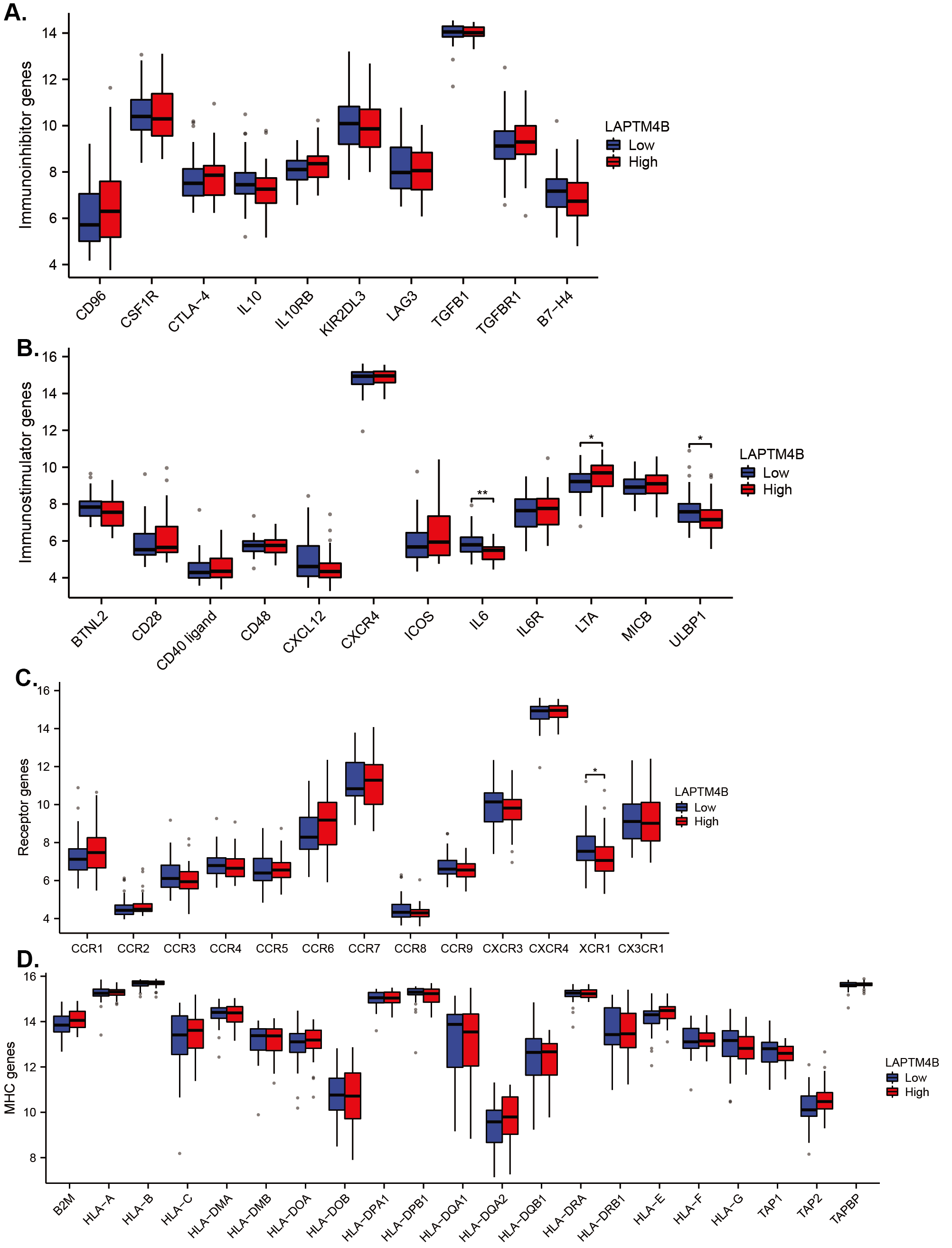


**Supplementary Figure 6. Association between LAPTM4B expression and immune related genes in ph+ B-ALL.** **(A)** The boxplots of immunoinhibitor genes in different LAPTM4B expression groups. **(B)** The boxplots of immunostimulator genes in different LAPTM4B expression groups. **(C)** The boxplots of receptor genes in different LAPTM4B expression groups. **(D)** The boxplots of MHC genes in different LAPTM4B expression groups.

**Supplemental Table 1. Details of diagnostic ROC for LAPTM4B in pan-cancer.**

| Tumor type | Tumor(n) | Normal(n) | AUC(CI) | cut-off | sensitivity | specificity | Positive predictive value | Negative predictive value | YI |
| --- | --- | --- | --- | --- | --- | --- | --- | --- | --- |
| ACC | 77 | 128 | 0.640 (0.552-0.728) | 5.541 | 0.506 | 0.805 | 0.609 | 0.730 | 0.311 |
| BLCA | 407 | 28 | 0.692 (0.628-0.756) | 5.703 | 0.602 | 0.893 | 0.988 | 0.134 | 0.495 |
| BRCA | 1099 | 292 | 0.821 (0.798-0.844) | 5.797 | 0.628 | 0.873 | 0.949 | 0.384 | 0.501 |
| CESC | 306 | 13 | 0.805 (0.732-0.878) | 6.091 | 0.676 | 0.923 | 0.995 | 0.108 | 0.600 |
| **CHOL-TCGA** | **36** | **9** | **0.988 (0.962-1.000)** | **5.272** | **1.000** | **0.972** | **0.900** | **1.000** | **0.972** |
| COAD | 290 | 349 | 0.890 (0.857-0.922) | 4.949 | 0.817 | 0.943 | 0.922 | 0.861 | 0.760 |
| DLBC | 47 | 444 | 0.823 (0.786-0.860) | 1.307 | 0.957 | 0.723 | 0.268 | 0.994 | 0.680 |
| ESAD | 80 | 10 | 0.764 (0.562-0.966) | 6.737 | 0.800 | 0.725 | 0.267 | 0.967 | 0.525 |
| **ESCA** | **182** | **666** | **0.960 (0.942-0.979)** | **5.397** | **0.907** | **0.932** | **0.786** | **0.973** | **0.839** |
| **GBM** | **166** | **1157** | **0.994 (0.989-1.000)** | **5.470** | **0.952** | **0.988** | **0.919** | **0.993** | **0.940** |
| **HNSC** | **502** | **44** | **0.908 (0.878-0.938)** | **6.869** | **0.977** | **0.741** | **0.249** | **0.997** | **0.718** |
| KICH | 66 | 53 | 0.617 (0.508-0.726) | 5.844 | 0.803 | 0.528 | 0.679 | 0.683 | 0.331 |
| KIRC | 531 | 100 | 0.747 (0.685-0.810) | 5.955 | 0.864 | 0.620 | 0.924 | 0.463 | 0.484 |
| KIRP | 289 | 60 | 0.685 (0.614-0.757) | 6.013 | 0.637 | 0.717 | 0.915 | 0.291 | 0.353 |
| **LAML** | **173** | **70** | **0.950 (0.919-0.981)** | **6.872** | **0.931** | **1.000** | **1.000** | **0.854** | **0.931** |
| **LGG** | **523** | **1152** | **0.994 (0.991-0.997)** | **5.310** | **0.950** | **0.978** | **0.952** | **0.977** | **0.929** |
| LIHC | 371 | 160 | 0.858 (0.825-0.890) | 4.093 | 0.752 | 0.906 | 0.949 | 0.612 | 0.658 |
| **LUAD** | **515** | **347** | **0.955 (0.942-0.967)** | **5.828** | **0.885** | **0.902** | **0.931** | **0.841** | **0.787** |
| **LUSC** | **498** | **338** | **0.959 (0.946-0.972)** | **5.913** | **0.853** | **0.962** | **0.970** | **0.817** | **0.815** |
| OSCC-TCGA | 329 | 32 | 0.896 (0.857-0.936) | 6.869 | 0.969 | 0.708 | 0.244 | 0.996 | 0.677 |
| **OV** | **427** | **88** | **0.994 (0.989-1.000)** | **6.333** | **0.965** | **1.000** | **1.000** | **0.854** | **0.965** |
| **PAAD** | **179** | **171** | **0.977 (0.962-0.991)** | **4.939** | **0.933** | **0.936** | **0.938** | **0.930** | **0.869** |
| PRAD | 496 | 152 | 0.704 (0.660-0.749) | 4.089 | 0.530 | 0.816 | 0.904 | 0.347 | 0.346 |
| **READ** | **93** | **318** | **0.967 (0.939-0.996)** | **4.855** | **0.935** | **0.943** | **0.829** | **0.980** | **0.879** |
| **SKCM** | **469** | **813** | **0.920 (0.900-0.939)** | **5.878** | **0.823** | **0.941** | **0.889** | **0.902** | **0.764** |
| **STAD** | **414** | **210** | **0.912 (0.889-0.936)** | **5.613** | **0.816** | **0.886** | **0.934** | **0.710** | **0.702** |
| **TGCT** | **154** | **165** | **0.962 (0.942-0.983)** | **6.783** | **0.851** | **0.994** | **0.992** | **0.877** | **0.845** |
| THCA | 512 | 338 | 0.614 (0.577-0.652) | 5.090 | 0.533 | 0.713 | 0.738 | 0.502 | 0.246 |
| **THYM** | **119** | **446** | **0.910 (0.885-0.935)** | **1.778** | **0.941** | **0.800** | **0.557** | **0.981** | **0.742** |
| **UCEC** | **181** | **101** | **0.913 (0.878-0.947)** | **6.345** | **0.823** | **0.901** | **0.937** | **0.740** | **0.724** |
| **UCS** | **57** | **78** | **0.972 (0.943-1.000)** | **5.855** | **0.965** | **0.897** | **0.873** | **0.972** | **0.862** |

Abbreviations:ROC: Receiver Operator Characteristic curve; AUC: Area Under Curve; CI: Confidence Interval; YI: Youden’s indx.
